# Metagenome-Assembled Genomes of Four Southern Ocean Archaea Harbor Multiple Genes Linked to Polyethylene Terephthalate and Polyhydroxybutyrate Plastic Degradation

**DOI:** 10.1128/mra.01098-22

**Published:** 2023-02-16

**Authors:** Yvonne Marvellous Akpudo, Oliver K. Bezuidt, Thulani P. Makhalanyane

**Affiliations:** a Microbiome@UP, Department of Biochemistry, Genetics, and Microbiology, University of Pretoria, Pretoria, South Africa; b South African Research Chair in Marine Microbiomics, Department of Biochemistry, Genetics, and Microbiology, University of Pretoria, Pretoria, South Africa; Portland State University

## Abstract

Here, we present four archaeal metagenome-assembled genomes (MAGs) (three *Thaumarchaeota* MAGs and one *Thermoplasmatota* MAG) from a polar upwelling zone in the Southern Ocean. These archaea harbor putative genes encoding enzymes such as polyethylene terephthalate (PET) hydrolases (PETases) and polyhydroxybutyrate (PHB) depolymerases, which are associated with microbial degradation of PET and PHB plastics.

## ANNOUNCEMENT

*Nitrosopumilus* and Marine Group (MG) II archaea affiliated with the phyla *Thaumarchaeota* and *Thermoplasmatota*, respectively, are widely distributed in shallow depths of the oceans (1–5). However, except for their capacity to sequester nitrogen and carbon in the oceans (1, 6, 7), their metabolic functions, including those linked to the biodegradation of plastic polymers, remain largely unclear. To reduce this knowledge deficit, we present four draft genomes of *Nitrosopumilus* and MG II archaea from the Southern Ocean. We further explore their metabolic potential linked to the degradation of polyethylene terephthalate (PET) and polyhydroxybutyrate (PHB).

Two water samples (10 L each) were collected from depths of approximately 5 m and 110 m at a polar upwelling zone (54.007°S, 0.011°E) during the SCALE (Southern oCean seAsonaL Experiment) expedition in October and November 2019. After sequential filtration with 0.5-μm glass-fiber and 0.2-μm polycarbonate filters, DNA was extracted from the latter size fraction using the DNeasy PowerWater kit (Qiagen, GmbH, Germany) as detailed in the manufacturer’s protocol. Sequencing libraries were generated using the Nextera XT kit (Illumina, Inc., San Diego, CA, USA) and sequenced using an Illumina NovaSeq S4 platform (2 × 150 bp) by Admera Biopharma Service Department (South Plainfield, NJ, USA). The Metagenome-ATLAS v2.4.4 workflow (8) was used to generate metagenome-assembled genomes (MAGs). ATLAS incorporates BBTools v38.87 for quality control (9) and metaSPAdes v3.14.1 for assembly of contigs (10). These assemblies were binned into MAGs using MetaBAT2 (11) and MaxBin2 (12). The resultant MAGs were refined and dereplicated with DASTool v1.1.2 (13) and dRep v2.5.4 (14), respectively. Completeness and contamination levels were computed using CheckM v1.1.3 (15). MAGs were uploaded onto KBase (16) for taxonomic assignments using GTDB-Tk v1.0.2 and the Genome Taxonomy Database (GTDB) reference release 89 (17). Also, the average nucleotide identity (ANI) values for these MAGs were calculated against each other using FastANI v1.32 (18). For this study, we selected four nonredundant archaeal MAGs, i.e., *Nitrosopumilus* and MG II, MG IIa-L1 (family *Poseidoniaceae*), MG IIb-O2, and MG IIb-O3 (family *Thalassoarchaeaceae*). The NCBI Prokaryotic Genome Annotation Pipeline (PGAP) v6.1 (19) was used for annotation of these MAGs, and the resultant data are presented in Table 1. To screen for plastic-degrading genes in these MAGs, protein-coding sequences were predicted with Prodigal v2.6.3 (20), and then HMMscan v3.1b2 (21) was implemented against profiles generated from cluster analysis of reference sequences obtained from the Plastics Microbial Biodegradation Database (PMBD) (22) using MCL v14-137 (23). Putative hits homologous to plastic-degrading genes were further analyzed using the HHpred interactive server with HHsuite v3.3.0 (24–26) at high similarity specification (>95% probability), which compared the secondary structures and domains of these predicted genes with references in the Protein Data Bank (PDB) mmCIF70 (27) and Pfam-A v34 (28) databases, respectively. Unless otherwise stated, default parameters were used for all software.

**TABLE 1:** General features of archaeal MAGs from a polar upwelling zone in the Southern Ocean

Genomic feature	Data for MAG:			
	S9-metabat_32 (*Nitrosopumilus*)	S10-maxbin_48 (MG IIa-L1)	S10-metabat_04 (MG IIb-O2)	S10-metabat_06 (MG IIb-O3)
No. of raw paired-end reads	63,532,776	53,101,891	53,101,891	53,101,891
Genome size (bp)	988,157	1,829,283	1,426,462	1,466,140
GC content (%)	30.98	46.22	38.01	35.85
No. of contigs	181	554	63	50
Minimum contig length (bp)	1,500	1,000	1,500	1,500
*N*_50_ (bp)	6,719	4,282	47,072	46,931
Completeness (%)^*a*^	91.13	66.67	82.31	75.73
Contamination (%)^*a*^	0.97	1.2	6.06	0
No. of predicted genes^*b*^	1,320	1,767	1,280	1,313
No. of coding sequences (with protein)^*b*^	1,278	1,719	1,241	1,272
No. of tRNAs^*b*^	36	36	29	35
No. of rRNAs^*b*^	1	3	2	3
No. of noncoding RNAs^*b*^	2	2	2	2
GenBank assembly accession no. for GTDB closest reference strain^*c*^	GCA_001437625.1	GCA_009937025.1	GCA_003672125.1	GCA_002496735.1
ANI (%)^*d*^	93.49	79.83	79.76	93.34
BioSample accession no.	SAMN23575727	SAMN23575728	SAMN23575729	SAMN23575730
GenBank accession no.	JAJPRB000000000	JAJPRC000000000	JAJPRD000000000	JAJPRE000000000
SRA accession no.	SRR17224336	SRR17224335	SRR17224335	SRR17224335

a Estimated with CheckM v1.1.3 (15).

b Numbers obtained from annotation with NCBI PGAP v6.1 (19).

c GTDB release 89 nearest reference strain determined with GTDB-Tk v1.0.2 (17).

d ANI between MAG and nearest GTDB reference strain estimated with FastANI v1.32 (18).

**FIG 1:**
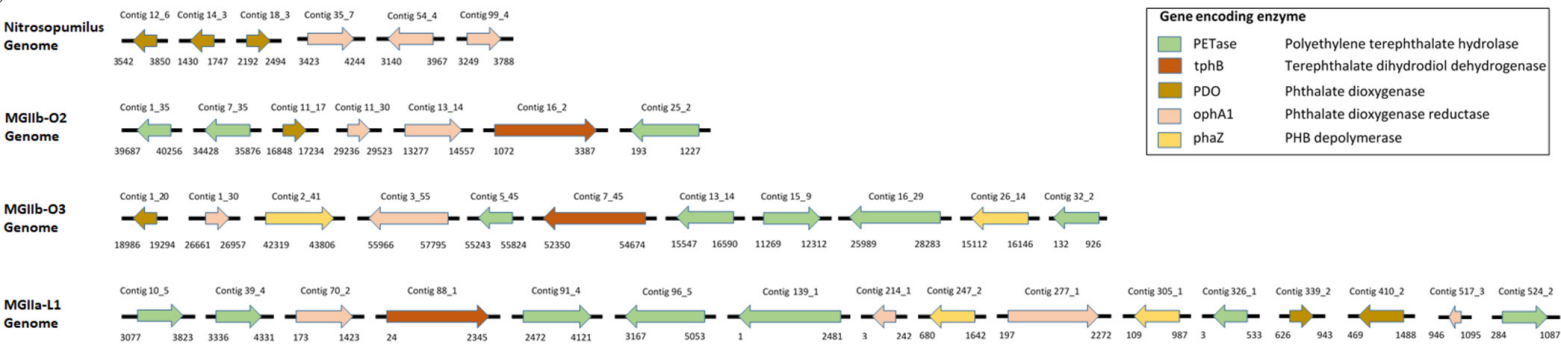
Predicted plastic-polymer-degrading genes, with their orientations indicated, for contiguous sequences of archaeal MAGs from a polar upwelling zone in the Southern Ocean. The predicted genes are shown as arrows, indicating contigs in ascending order. The secondary structure and domains were compared to references in the PDB mmCIF70 and Pfam-A databases, respectively, using HHpred, which yielded high similarity of >95% probability. Labels above the arrows represent contig identification numbers, as specified in the protein-coding sequence from Prodigal. The numbers before and after the underscore represent the sequence position in the genome and the gene position within the contig, respectively. Labels below the arrows show the distance between the annotated start and end, equated as the gene size and represented by the relative length of each arrow.

As shown in Fig. 1, this analysis resulted in the prediction of multiple genes encoding enzymes that catalyze PET (29, 30) and PHB (31) plastic degradation in MG II genomes. The *Nitrosopumilus* genome harbored only putative phthalate dioxygenase (PDO) and reductase (*ophA1*) genes, which are associated with the degradation of the plasticizer phthalate (7, 32).

### Data availability.

Raw sequence reads (SRA accession numbers SRR17224336 and SRR17224335) are available in the NCBI GenBank database under BioProject accession number PRJNA785751. The archaeal MAGs were assigned BioSample numbers SAMN23575727 to SAMN23575730, as well as GenBank accession numbers JAJPRB000000000, JAJPRC000000000, JAJPRD000000000, and JAJPRE000000000. The versions described in this paper are the first versions (JAJPRB010000000, JAJPRC010000000, JAJPRD010000000, and JAJPRE010000000).
